# Supplementary material for: Cervical Fluids Are a Source of Protein Biomarkers for Early, Non-Invasive Endometrial Cancer Diagnosis
Source: Cancers (Basel). 2023 Jan 31;15(3):911. doi: 10.3390/cancers15030911 (PMC9913506; doi:10.3390/cancers15030911)
Supplement: Supplementary file 1 [file cancers-15-00911-s001.zip › Suppl_Table1.1.pdf]

Protein Name	Peptide Sequence	Precursor Mz	Precursor Charge
sp|P17931|LEG3_HUMAN	IALDFQR	431.742702	2
sp|P17931|LEG3_HUMAN	IALDFQR	436.746837	2
sp|P17931|LEG3_HUMAN	GNDVAFHFNPR	425.207266	3
sp|P17931|LEG3_HUMAN	GNDVAFHFNPR	428.543355	3
sp|P10451|OSTP_HUMAN	AIPVAQDLNAPSDWDSR	927.952671	2
sp|P10451|OSTP_HUMAN	AIPVAQDLNAPSDWDSR	932.956806	2
sp|P10451|OSTP_HUMAN	ANDESNEHSDVIDSQELSK	706.313693	3
sp|P10451|OSTP_HUMAN	ANDESNEHSDVIDSQELSK	708.985092	3
sp|P35222|CTNB1_HUMAN	LLNDEDQVVVNK	693.367181	2
sp|P35222|CTNB1_HUMAN	LLNDEDQVVVNK	697.37428	2
sp|P35222|CTNB1_HUMAN	LVQLLVR	420.786913	2
sp|P35222|CTNB1_HUMAN	LVQLLVR	425.791047	2
sp|P30041|PRDX6_HUMAN	DFTPVCTTELGR	698.332284	2
sp|P30041|PRDX6_HUMAN	DFTPVCTTELGR	703.336418	2
sp|P30041|PRDX6_HUMAN	LSILYPATTGR	596.340238	2
sp|P30041|PRDX6_HUMAN	LSILYPATTGR	601.344372	2
sp|O00299|CLIC1_HUMAN	LAALNPESNTAGLDIFAK	922.991265	2
sp|O00299|CLIC1_HUMAN	LAALNPESNTAGLDIFAK	926.998365	2
sp|O00299|CLIC1_HUMAN	NSNPALNDNLEK	664.82568	2
sp|O00299|CLIC1_HUMAN	NSNPALNDNLEK	668.832779	2
sp|P14618|KPYM_HUMAN	NTGIICTIGPASR	680.356093	2
sp|P14618|KPYM_HUMAN	NTGIICTIGPASR	685.360228	2
sp|P14618|KPYM_HUMAN	APIIAVTR	420.76872	2
sp|P14618|KPYM_HUMAN	APIIAVTR	425.772854	2
sp|P07355|ANXA2_HUMAN	GVDEVTIVNILTNR	771.927937	2
sp|P07355|ANXA2_HUMAN	GVDEVTIVNILTNR	776.932071	2
sp|P07355|ANXA2_HUMAN	QDIAFAYQR	556.28018	2
sp|P07355|ANXA2_HUMAN	QDIAFAYQR	561.284315	2
sp|P15941|MUC1_HUMAN	QGGFLGLSNIK	567.319305	2
sp|P15941|MUC1_HUMAN	QGGFLGLSNIK	571.326405	2
sp|P15941|MUC1_HUMAN	EGTINVHDVETQFNQYK	674.656954	3
sp|P15941|MUC1_HUMAN	EGTINVHDVETQFNQYK	677.328354	3
sp|P55060|XPO2_HUMAN	ANIVHLMLSSPEQIQK	603.329344	3
sp|P55060|XPO2_HUMAN	ANIVHLMLSSPEQIQK	606.000744	3
sp|P55060|XPO2_HUMAN	LLQTDDEEEAGLLELLK	965.006778	2
sp|P55060|XPO2_HUMAN	LLQTDDEEEAGLLELLK	969.013878	2
sp|P50454|SERPH_HUMAN	GVVEVTHDLQK	408.890943	3
sp|P50454|SERPH_HUMAN	GVVEVTHDLQK	411.562343	3
sp|P50454|SERPH_HUMAN	DTQSGSLLFIGR	647.343509	2
sp|P50454|SERPH_HUMAN	DTQSGSLLFIGR	652.347643	2
sp|P16035|TIMP2_HUMAN	EVDSGNDIYGNPIK	760.865002	2
sp|P16035|TIMP2_HUMAN	EVDSGNDIYGNPIK	764.872102	2
sp|P09382|LEG1_HUMAN	SFVLNLGK	439.260728	2
sp|P09382|LEG1_HUMAN	SFVLNLGK	443.267827	2
sp|P09382|LEG1_HUMAN	LPDGYEFK	484.739825	2
sp|P09382|LEG1_HUMAN	LPDGYEFK	488.746925	2
sp|Q13938|CAYP1_HUMAN	EAVIAAAFAK	495.784567	2
sp|Q13938|CAYP1_HUMAN	EAVIAAAFAK	499.791666	2
sp|Q13938|CAYP1_HUMAN	SGDGVVTVDDLR	616.809499	2
sp|Q13938|CAYP1_HUMAN	SGDGVVTVDDLR	621.813633	2
sp|P42574|CASP3_HUMAN	SGTDVDAANLR	559.775459	2
sp|P42574|CASP3_HUMAN	SGTDVDAANLR	564.779593	2
sp|P16949|STMN1_HUMAN	SHEAEVLK	456.742899	2
sp|P16949|STMN1_HUMAN	SHEAEVLK	460.749999	2
sp|P16949|STMN1_HUMAN	AIEENNNFSK	583.277834	2
sp|P16949|STMN1_HUMAN	AIEENNNFSK	587.284934	2
sp|P14780|MMP9_HUMAN	SLGPALLLLQK	576.871174	2
sp|P14780|MMP9_HUMAN	SLGPALLLLQK	580.878273	2
sp|P14780|MMP9_HUMAN	AFALWSAVTPLTFTR	840.95904	2
sp|P14780|MMP9_HUMAN	AFALWSAVTPLTFTR	845.963174	2
sp|P08670|VIME_HUMAN	ILLAELEQLK	585.360639	2
sp|P08670|VIME_HUMAN	ILLAELEQLK	589.367738	2
sp|P08670|VIME_HUMAN	ISLPLPNFSSLNLR	785.951215	2
sp|P08670|VIME_HUMAN	ISLPLPNFSSLNLR	790.955349	2
sp|P16070|CD44_HUMAN	TEAADLCK	454.213117	2
