# Supplementary material for: Cervical Fluids Are a Source of Protein Biomarkers for Early, Non-Invasive Endometrial Cancer Diagnosis
Source: Cancers (Basel). 2023 Jan 31;15(3):911. doi: 10.3390/cancers15030911 (PMC9913506; doi:10.3390/cancers15030911)
Supplement: Supplementary file 1 [file cancers-15-00911-s001.zip › Suppl_Table1.2.pdf]

sp|P16070|CD44_HUMAN	TEAADLCK	458.220217	2
sp|P15328|FOLR1_HUMAN	TELLNVCMNAK	646.820498	2
sp|P15328|FOLR1_HUMAN	TELLNVCMNAK	650.827597	2
sp|P15328|FOLR1_HUMAN	FNWNHCGEMAPACK	574.570893	3
sp|P15328|FOLR1_HUMAN	FNWNHCGEMAPACK	577.242293	3
sp|O43852|CALU_HUMAN	TFDQLTPEESK	647.811707	2
sp|O43852|CALU_HUMAN	TFDQLTPEESK	651.818806	2
sp|P09603|CSF1_HUMAN	AFLLVQDIMEDTMR	841.418033	2
sp|P09603|CSF1_HUMAN	AFLLVQDIMEDTMR	846.422168	2
sp|P09603|CSF1_HUMAN	TFYETPLQLLEK	741.39795	2
sp|P09603|CSF1_HUMAN	TFYETPLQLLEK	745.405049	2
sp|Q06830|PRDX1_HUMAN	ADEGISFR	447.719424	2
sp|Q06830|PRDX1_HUMAN	ADEGISFR	452.723558	2
sp|Q06830|PRDX1_HUMAN	LVQAFQFTDK	598.819138	2
sp|Q06830|PRDX1_HUMAN	LVQAFQFTDK	602.826238	2
sp|O75556|SG2A1_HUMAN	TINSDISIPEYK	690.356282	2
sp|O75556|SG2A1_HUMAN	TINSDISIPEYK	694.363381	2
sp|O75556|SG2A1_HUMAN	ELLQEFIDSDAAAEAMGK	646.643546	3
sp|O75556|SG2A1_HUMAN	ELLQEFIDSDAAAEAMGK	649.314945	3
sp|P22626|ROA2_HUMAN	TLETVPLER	529.298038	2
sp|P22626|ROA2_HUMAN	TLETVPLER	534.302173	2
sp|P22626|ROA2_HUMAN	GGNFGFGDSR	507.225405	2
sp|P22626|ROA2_HUMAN	GGNFGFGDSR	512.229539	2
sp|P09211|GSTP1_HUMAN	ASCLYGQLPK	568.792066	2
sp|P09211|GSTP1_HUMAN	ASCLYGQLPK	572.799166	2
sp|P09211|GSTP1_HUMAN	TLGLYGK	376.221071	2
sp|P09211|GSTP1_HUMAN	TLGLYGK	380.228171	2
sp|Q01995|TAGL_HUMAN	TLMALGSLAVTK	602.852124	2
sp|Q01995|TAGL_HUMAN	TLMALGSLAVTK	606.859223	2
sp|Q01995|TAGL_HUMAN	EFTESQLQEGK	648.309331	2
sp|Q01995|TAGL_HUMAN	EFTESQLQEGK	652.316431	2
sp|P00441|SODC_HUMAN	AVCVLK	345.204367	2
sp|P00441|SODC_HUMAN	AVCVLK	349.211466	2
sp|P00441|SODC_HUMAN	TLVVHEK	413.245078	2
sp|P00441|SODC_HUMAN	TLVVHEK	417.252177	2
sp|P04083|ANXA1_HUMAN	GGPGSAVSPYPTFNPSSDVAALHK	786.057118	3
sp|P04083|ANXA1_HUMAN	GGPGSAVSPYPTFNPSSDVAALHK	788.728517	3
sp|P04083|ANXA1_HUMAN	DITSDTSGDFR	607.270206	2
sp|P04083|ANXA1_HUMAN	DITSDTSGDFR	612.274341	2
sp|Q01469|FABP5_HUMAN	ELGVGIALR	464.284734	2
sp|Q01469|FABP5_HUMAN	ELGVGIALR	469.288869	2
sp|Q01469|FABP5_HUMAN	LVVECVMNNVTCTR	847.904767	2
sp|Q01469|FABP5_HUMAN	LVVECVMNNVTCTR	852.908902	2
sp|P08253|MMP2_HUMAN	IDAVYEAPQEEK	696.338089	2
sp|P08253|MMP2_HUMAN	IDAVYEAPQEEK	700.345188	2
sp|P08253|MMP2_HUMAN	VDAAFNWSK	519.256174	2
sp|P08253|MMP2_HUMAN	VDAAFNWSK	523.263273	2
sp|P07237|PDIA1_HUMAN	ALAPEYAK	431.737085	2
sp|P07237|PDIA1_HUMAN	ALAPEYAK	435.744185	2
sp|P07237|PDIA1_HUMAN	ILEFFGLK	483.786578	2
sp|P07237|PDIA1_HUMAN	ILEFFGLK	487.793678	2
sp|P12830|CADH1_HUMAN	NLVQIK	357.729063	2
sp|P12830|CADH1_HUMAN	NLVQIK	361.736163	2
sp|P12830|CADH1_HUMAN	VFYSITGQGADTPPVGVFIIER	789.417875	3
sp|P12830|CADH1_HUMAN	VFYSITGQGADTPPVGVFIIER	792.753965	3
sp|P61604|CH10_HUMAN	FLPLFDR	454.255445	2
sp|P61604|CH10_HUMAN	FLPLFDR	459.25958	2
sp|P61604|CH10_HUMAN	VLLPEYGGTK	538.802957	2
sp|P61604|CH10_HUMAN	VLLPEYGGTK	542.810056	2
sp|P30086|PEBP1_HUMAN	VLTPTQVK	443.273835	2
sp|P30086|PEBP1_HUMAN	VLTPTQVK	447.280935	2
sp|P30086|PEBP1_HUMAN	LYEQLSGK	469.2531	2
sp|P30086|PEBP1_HUMAN	LYEQLSGK	473.260199	2
sp|P09466|PAEP_HUMAN	VLVEDDEIMQGFIR	832.42163	2
sp|P09466|PAEP_HUMAN	VLVEDDEIMQGFIR	837.425764	2
sp|P80188|NGAL_HUMAN	VPLQQNFQDNQFQGK	597.632192	3
