# Supplementary material for: Cervical Fluids Are a Source of Protein Biomarkers for Early, Non-Invasive Endometrial Cancer Diagnosis
Source: Cancers (Basel). 2023 Jan 31;15(3):911. doi: 10.3390/cancers15030911 (PMC9913506; doi:10.3390/cancers15030911)
Supplement: Supplementary file 1 [file cancers-15-00911-s001.zip › Suppl_Table1.3.pdf]

sp|P80188|NGAL_HUMAN	VPLQQNFQDNQFQGK	600.303591	3
sp|P80188|NGAL_HUMAN	ELTSELK	410.22655	2
sp|P80188|NGAL_HUMAN	ELTSELK	414.23365	2
sp|Q14508|WFDC2_HUMAN	EGSCPQVNINFPQLGLCR	697.003303	3
sp|Q14508|WFDC2_HUMAN	EGSCPQVNINFPQLGLCR	700.339392	3
sp|Q14508|WFDC2_HUMAN	VSCVTPNF	462.218203	2
sp|Q14508|WFDC2_HUMAN	VSCVTPNF	467.231817	2
sp|P62937|PPIA_HUMAN	VSFELFADK	528.274032	2
sp|P62937|PPIA_HUMAN	VSFELFADK	532.281132	2
sp|P62937|PPIA_HUMAN	FEDENFILK	577.790046	2
sp|P62937|PPIA_HUMAN	FEDENFILK	581.797146	2
sp|P04792|HSPB1_HUMAN	LFDQAFGLPR	582.314023	2
sp|P04792|HSPB1_HUMAN	LFDQAFGLPR	587.318157	2
sp|P04792|HSPB1_HUMAN	LATQSNEITIPVTFESR	953.499455	2
sp|P04792|HSPB1_HUMAN	LATQSNEITIPVTFESR	958.503589	2
sp|P07339|CATD_HUMAN	VSTLPAITLK	521.828974	2
sp|P07339|CATD_HUMAN	VSTLPAITLK	525.836074	2
sp|P07339|CATD_HUMAN	VGFAEAAR	410.719227	2
sp|P07339|CATD_HUMAN	VGFAEAAR	415.723361	2
sp|P11166|GTR1_HUMAN	TFDEIASGFR	571.77747	2
sp|P11166|GTR1_HUMAN	TFDEIASGFR	576.781605	2
sp|P00338|LDHA_HUMAN	LVIITAGAR	457.295102	2
sp|P00338|LDHA_HUMAN	LVIITAGAR	462.299236	2
sp|P00338|LDHA_HUMAN	VTLTSEEEAR	567.785492	2
sp|P00338|LDHA_HUMAN	VTLTSEEEAR	572.789627	2
sp|P60174|TPIS_HUMAN	VVLAYEPVWAIGTGK	801.948141	2
sp|P60174|TPIS_HUMAN	VVLAYEPVWAIGTGK	805.95524	2
sp|P60174|TPIS_HUMAN	SNVSDAVAQSTR	617.804748	2
sp|P60174|TPIS_HUMAN	SNVSDAVAQSTR	622.808882	2
sp|P05164|PERM_HUMAN	IANVFTNAFR	576.811647	2
sp|P05164|PERM_HUMAN	IANVFTNAFR	581.815782	2
sp|P05164|PERM_HUMAN	VVLEGGIDPILR	640.88227	2
sp|P05164|PERM_HUMAN	VVLEGGIDPILR	645.886404	2
sp|P14174|MIF_HUMAN	LLCGLLAER	522.797151	2
sp|P14174|MIF_HUMAN	LLCGLLAER	527.801286	2
sp|P14174|MIF_HUMAN	VYINYYDMNAANVGWNNSTFA	809.695191	3
sp|P14174|MIF_HUMAN	VYINYYDMNAANVGWNNSTFA	813.037601	3
sp|P01833|PIGR_HUMAN	GGCITLISSEGYVSSK	829.40872	2
sp|P01833|PIGR_HUMAN	GGCITLISSEGYVSSK	833.415819	2
sp|P01833|PIGR_HUMAN	VYTVDLGR	461.753267	2
sp|P01833|PIGR_HUMAN	VYTVDLGR	466.757401	2
sp|P05787|K2C8_HUMAN	WSLLQQQK	515.787641	2
sp|P05787|K2C8_HUMAN	WSLLQQQK	519.79474	2
sp|P05787|K2C8_HUMAN	LSELEAALQR	565.31422	2
sp|P05787|K2C8_HUMAN	LSELEAALQR	570.318354	2
sp|O43278|SPIT1_HUMAN	WYYDPTEQICK	751.834659	2
sp|O43278|SPIT1_HUMAN	WYYDPTEQICK	755.841759	2
sp|O43278|SPIT1_HUMAN	SFVYGGCLGNK	601.287148	2
sp|O43278|SPIT1_HUMAN	SFVYGGCLGNK	605.294247	2
sp|P06733|ENOA_HUMAN	TIAPALVSK	450.28166	2
sp|P06733|ENOA_HUMAN	TIAPALVSK	454.28876	2
sp|P06733|ENOA_HUMAN	YISPDQLADLYK	713.366649	2
sp|P06733|ENOA_HUMAN	YISPDQLADLYK	717.373749	2
sp|Q16658|FSCN1_HUMAN	FLIVAHDDGR	381.536612	3
sp|Q16658|FSCN1_HUMAN	FLIVAHDDGR	384.872701	3
sp|Q16658|FSCN1_HUMAN	YLAPSGPSGTLK	595.82442	2
sp|Q16658|FSCN1_HUMAN	YLAPSGPSGTLK	599.83152	2
sp|P43490|NAMPT_HUMAN	YDGHLPIEIK	395.548517	3
sp|P43490|NAMPT_HUMAN	YDGHLPIEIK	398.219917	3
sp|P43490|NAMPT_HUMAN	YLLETSGNLDGLEYK	857.930342	2
sp|P43490|NAMPT_HUMAN	YLLETSGNLDGLEYK	861.937442	2
sp|P40121|CAPG_HUMAN	YQEGGVESAFHK	451.214086	3
sp|P40121|CAPG_HUMAN	YQEGGVESAFHK	453.885486	3
sp|P40121|CAPG_HUMAN	EGNPEEDLTADK	659.293878	2
sp|P40121|CAPG_HUMAN	EGNPEEDLTADK	663.300978	2
