# Supplementary material for: Cervical Fluids Are a Source of Protein Biomarkers for Early, Non-Invasive Endometrial Cancer Diagnosis
Source: Cancers (Basel). 2023 Jan 31;15(3):911. doi: 10.3390/cancers15030911 (PMC9913506; doi:10.3390/cancers15030911)
Supplement: Supplementary file 1 [file cancers-15-00911-s001.zip › Suppl_Table1.pdf]

Protein Name	Peptide Sequence	Precursor Mz	Precursor Charge
sp|P17931|LEG3_HUMAN	IALDFQR	431.742702	2
sp|P17931|LEG3_HUMAN	IALDFQR	436.746837	2
sp|P17931|LEG3_HUMAN	GNDVAFHFNPR	425.207266	3
sp|P17931|LEG3_HUMAN	GNDVAFHFNPR	428.543355	3
sp|P10451|OSTP_HUMAN	AIPVAQDLNAPSDWDSR	927.952671	2
sp|P10451|OSTP_HUMAN	AIPVAQDLNAPSDWDSR	932.956806	2
sp|P10451|OSTP_HUMAN	ANDESNEHSDVIDSQELSK	706.313693	3
sp|P10451|OSTP_HUMAN	ANDESNEHSDVIDSQELSK	708.985092	3
sp|P35222|CTNB1_HUMAN	LLNDEDQVVVNK	693.367181	2
sp|P35222|CTNB1_HUMAN	LLNDEDQVVVNK	697.37428	2
sp|P35222|CTNB1_HUMAN	LVQLLVR	420.786913	2
sp|P35222|CTNB1_HUMAN	LVQLLVR	425.791047	2
sp|P30041|PRDX6_HUMAN	DFTPVCTTELGR	698.332284	2
sp|P30041|PRDX6_HUMAN	DFTPVCTTELGR	703.336418	2
sp|P30041|PRDX6_HUMAN	LSILYPATTGR	596.340238	2
sp|P30041|PRDX6_HUMAN	LSILYPATTGR	601.344372	2
sp|O00299|CLIC1_HUMAN	LAALNPESNTAGLDIFAK	922.991265	2
sp|O00299|CLIC1_HUMAN	LAALNPESNTAGLDIFAK	926.998365	2
sp|O00299|CLIC1_HUMAN	NSNPALNDNLEK	664.82568	2
sp|O00299|CLIC1_HUMAN	NSNPALNDNLEK	668.832779	2
sp|P14618|KPYM_HUMAN	NTGIICTIGPASR	680.356093	2
sp|P14618|KPYM_HUMAN	NTGIICTIGPASR	685.360228	2
sp|P14618|KPYM_HUMAN	APIIAVTR	420.76872	2
sp|P14618|KPYM_HUMAN	APIIAVTR	425.772854	2
sp|P07355|ANXA2_HUMAN	GVDEVTIVNILTNR	771.927937	2
sp|P07355|ANXA2_HUMAN	GVDEVTIVNILTNR	776.932071	2
sp|P07355|ANXA2_HUMAN	QDIAFAYQR	556.28018	2
sp|P07355|ANXA2_HUMAN	QDIAFAYQR	561.284315	2
sp|P15941|MUC1_HUMAN	QGGFLGLSNIK	567.319305	2
sp|P15941|MUC1_HUMAN	QGGFLGLSNIK	571.326405	2
sp|P15941|MUC1_HUMAN	EGTINVHDVETQFNQYK	674.656954	3
sp|P15941|MUC1_HUMAN	EGTINVHDVETQFNQYK	677.328354	3
sp|P55060|XPO2_HUMAN	ANIVHLMLSSPEQIQK	603.329344	3
sp|P55060|XPO2_HUMAN	ANIVHLMLSSPEQIQK	606.000744	3
sp|P55060|XPO2_HUMAN	LLQTDDEEEAGLLELLK	965.006778	2
sp|P55060|XPO2_HUMAN	LLQTDDEEEAGLLELLK	969.013878	2
sp|P50454|SERPH_HUMAN	GVVEVTHDLQK	408.890943	3
sp|P50454|SERPH_HUMAN	GVVEVTHDLQK	411.562343	3
sp|P50454|SERPH_HUMAN	DTQSGSLLFIGR	647.343509	2
sp|P50454|SERPH_HUMAN	DTQSGSLLFIGR	652.347643	2
sp|P16035|TIMP2_HUMAN	EVDSGNDIYGNPIK	760.865002	2
sp|P16035|TIMP2_HUMAN	EVDSGNDIYGNPIK	764.872102	2
sp|P09382|LEG1_HUMAN	SFVLNLGK	439.260728	2
sp|P09382|LEG1_HUMAN	SFVLNLGK	443.267827	2
sp|P09382|LEG1_HUMAN	LPDGYEFK	484.739825	2
sp|P09382|LEG1_HUMAN	LPDGYEFK	488.746925	2
sp|Q13938|CAYP1_HUMAN	EAVIAAAFAK	495.784567	2
sp|Q13938|CAYP1_HUMAN	EAVIAAAFAK	499.791666	2
sp|Q13938|CAYP1_HUMAN	SGDGVVTVDDLR	616.809499	2
sp|Q13938|CAYP1_HUMAN	SGDGVVTVDDLR	621.813633	2
sp|P42574|CASP3_HUMAN	SGTDVDAANLR	559.775459	2
sp|P42574|CASP3_HUMAN	SGTDVDAANLR	564.779593	2
sp|P16949|STMN1_HUMAN	SHEAEVLK	456.742899	2
sp|P16949|STMN1_HUMAN	SHEAEVLK	460.749999	2
sp|P16949|STMN1_HUMAN	AIEENNNFSK	583.277834	2
sp|P16949|STMN1_HUMAN	AIEENNNFSK	587.284934	2
sp|P14780|MMP9_HUMAN	SLGPALLLLQK	576.871174	2
sp|P14780|MMP9_HUMAN	SLGPALLLLQK	580.878273	2
sp|P14780|MMP9_HUMAN	AFALWSAVTPLTFTR	840.95904	2
sp|P14780|MMP9_HUMAN	AFALWSAVTPLTFTR	845.963174	2
sp|P08670|VIME_HUMAN	ILLAELEQLK	585.360639	2
sp|P08670|VIME_HUMAN	ILLAELEQLK	589.367738	2
sp|P08670|VIME_HUMAN	ISLPLPNFSSLNLR	785.951215	2
sp|P08670|VIME_HUMAN	ISLPLPNFSSLNLR	790.955349	2
sp|P16070|CD44_HUMAN	TEAADLCK	454.213117	2

sp|P16070|CD44_HUMAN	TEAADLCK	458.220217	2
sp|P15328|FOLR1_HUMAN	TELLNVCMNAK	646.820498	2
sp|P15328|FOLR1_HUMAN	TELLNVCMNAK	650.827597	2
sp|P15328|FOLR1_HUMAN	FNWNHCGEMAPACK	574.570893	3
sp|P15328|FOLR1_HUMAN	FNWNHCGEMAPACK	577.242293	3
sp|O43852|CALU_HUMAN	TFDQLTPEESK	647.811707	2
sp|O43852|CALU_HUMAN	TFDQLTPEESK	651.818806	2
sp|P09603|CSF1_HUMAN	AFLLVQDIMEDTMR	841.418033	2
sp|P09603|CSF1_HUMAN	AFLLVQDIMEDTMR	846.422168	2
sp|P09603|CSF1_HUMAN	TFYETPLQLLEK	741.39795	2
sp|P09603|CSF1_HUMAN	TFYETPLQLLEK	745.405049	2
sp|Q06830|PRDX1_HUMAN	ADEGISFR	447.719424	2
sp|Q06830|PRDX1_HUMAN	ADEGISFR	452.723558	2
sp|Q06830|PRDX1_HUMAN	LVQAFQFTDK	598.819138	2
sp|Q06830|PRDX1_HUMAN	LVQAFQFTDK	602.826238	2
sp|O75556|SG2A1_HUMAN	TINSDISIPEYK	690.356282	2
sp|O75556|SG2A1_HUMAN	TINSDISIPEYK	694.363381	2
sp|O75556|SG2A1_HUMAN	ELLQEFIDSDAAAEAMGK	646.643546	3
sp|O75556|SG2A1_HUMAN	ELLQEFIDSDAAAEAMGK	649.314945	3
sp|P22626|ROA2_HUMAN	TLETVPLER	529.298038	2
sp|P22626|ROA2_HUMAN	TLETVPLER	534.302173	2
sp|P22626|ROA2_HUMAN	GGNFGFGDSR	507.225405	2
sp|P22626|ROA2_HUMAN	GGNFGFGDSR	512.229539	2
sp|P09211|GSTP1_HUMAN	ASCLYGQLPK	568.792066	2
sp|P09211|GSTP1_HUMAN	ASCLYGQLPK	572.799166	2
sp|P09211|GSTP1_HUMAN	TLGLYGK	376.221071	2
sp|P09211|GSTP1_HUMAN	TLGLYGK	380.228171	2
sp|Q01995|TAGL_HUMAN	TLMALGSLAVTK	602.852124	2
sp|Q01995|TAGL_HUMAN	TLMALGSLAVTK	606.859223	2
sp|Q01995|TAGL_HUMAN	EFTESQLQEGK	648.309331	2
sp|Q01995|TAGL_HUMAN	EFTESQLQEGK	652.316431	2
sp|P00441|SODC_HUMAN	AVCVLK	345.204367	2
sp|P00441|SODC_HUMAN	AVCVLK	349.211466	2
sp|P00441|SODC_HUMAN	TLVVHEK	413.245078	2
sp|P00441|SODC_HUMAN	TLVVHEK	417.252177	2
sp|P04083|ANXA1_HUMAN	GGPGSAVSPYPTFNPSSDVAALHK	786.057118	3
sp|P04083|ANXA1_HUMAN	GGPGSAVSPYPTFNPSSDVAALHK	788.728517	3
sp|P04083|ANXA1_HUMAN	DITSDTSGDFR	607.270206	2
sp|P04083|ANXA1_HUMAN	DITSDTSGDFR	612.274341	2
sp|Q01469|FABP5_HUMAN	ELGVGIALR	464.284734	2
sp|Q01469|FABP5_HUMAN	ELGVGIALR	469.288869	2
sp|Q01469|FABP5_HUMAN	LVVECVMNNVTCTR	847.904767	2
sp|Q01469|FABP5_HUMAN	LVVECVMNNVTCTR	852.908902	2
sp|P08253|MMP2_HUMAN	IDAVYEAPQEEK	696.338089	2
sp|P08253|MMP2_HUMAN	IDAVYEAPQEEK	700.345188	2
sp|P08253|MMP2_HUMAN	VDAAFNWSK	519.256174	2
sp|P08253|MMP2_HUMAN	VDAAFNWSK	523.263273	2
sp|P07237|PDIA1_HUMAN	ALAPEYAK	431.737085	2
sp|P07237|PDIA1_HUMAN	ALAPEYAK	435.744185	2
sp|P07237|PDIA1_HUMAN	ILEFFGLK	483.786578	2
sp|P07237|PDIA1_HUMAN	ILEFFGLK	487.793678	2
sp|P12830|CADH1_HUMAN	NLVQIK	357.729063	2
sp|P12830|CADH1_HUMAN	NLVQIK	361.736163	2
sp|P12830|CADH1_HUMAN	VFYSITGQGADTPPVGVFIIER	789.417875	3
sp|P12830|CADH1_HUMAN	VFYSITGQGADTPPVGVFIIER	792.753965	3
sp|P61604|CH10_HUMAN	FLPLFDR	454.255445	2
sp|P61604|CH10_HUMAN	FLPLFDR	459.25958	2
sp|P61604|CH10_HUMAN	VLLPEYGGTK	538.802957	2
sp|P61604|CH10_HUMAN	VLLPEYGGTK	542.810056	2
sp|P30086|PEBP1_HUMAN	VLTPTQVK	443.273835	2
sp|P30086|PEBP1_HUMAN	VLTPTQVK	447.280935	2
sp|P30086|PEBP1_HUMAN	LYEQLSGK	469.2531	2
sp|P30086|PEBP1_HUMAN	LYEQLSGK	473.260199	2
sp|P09466|PAEP_HUMAN	VLVEDDEIMQGFIR	832.42163	2
sp|P09466|PAEP_HUMAN	VLVEDDEIMQGFIR	837.425764	2
sp|P80188|NGAL_HUMAN	VPLQQNFQDNQFQGK	597.632192	3

sp|P80188|NGAL_HUMAN	VPLQQNFQDNQFQGK	600.303591	3
sp|P80188|NGAL_HUMAN	ELTSELK	410.22655	2
sp|P80188|NGAL_HUMAN	ELTSELK	414.23365	2
sp|Q14508|WFDC2_HUMAN	EGSCPQVNINFPQLGLCR	697.003303	3
sp|Q14508|WFDC2_HUMAN	EGSCPQVNINFPQLGLCR	700.339392	3
sp|Q14508|WFDC2_HUMAN	VSCVTPNF	462.218203	2
sp|Q14508|WFDC2_HUMAN	VSCVTPNF	467.231817	2
sp|P62937|PPIA_HUMAN	VSFELFADK	528.274032	2
sp|P62937|PPIA_HUMAN	VSFELFADK	532.281132	2
sp|P62937|PPIA_HUMAN	FEDENFILK	577.790046	2
sp|P62937|PPIA_HUMAN	FEDENFILK	581.797146	2
sp|P04792|HSPB1_HUMAN	LFDQAFGLPR	582.314023	2
sp|P04792|HSPB1_HUMAN	LFDQAFGLPR	587.318157	2
sp|P04792|HSPB1_HUMAN	LATQSNEITIPVTFESR	953.499455	2
sp|P04792|HSPB1_HUMAN	LATQSNEITIPVTFESR	958.503589	2
sp|P07339|CATD_HUMAN	VSTLPAITLK	521.828974	2
sp|P07339|CATD_HUMAN	VSTLPAITLK	525.836074	2
sp|P07339|CATD_HUMAN	VGFAEAAR	410.719227	2
sp|P07339|CATD_HUMAN	VGFAEAAR	415.723361	2
sp|P11166|GTR1_HUMAN	TFDEIASGFR	571.77747	2
sp|P11166|GTR1_HUMAN	TFDEIASGFR	576.781605	2
sp|P00338|LDHA_HUMAN	LVIITAGAR	457.295102	2
sp|P00338|LDHA_HUMAN	LVIITAGAR	462.299236	2
sp|P00338|LDHA_HUMAN	VTLTSEEEAR	567.785492	2
sp|P00338|LDHA_HUMAN	VTLTSEEEAR	572.789627	2
sp|P60174|TPIS_HUMAN	VVLAYEPVWAIGTGK	801.948141	2
sp|P60174|TPIS_HUMAN	VVLAYEPVWAIGTGK	805.95524	2
sp|P60174|TPIS_HUMAN	SNVSDAVAQSTR	617.804748	2
sp|P60174|TPIS_HUMAN	SNVSDAVAQSTR	622.808882	2
sp|P05164|PERM_HUMAN	IANVFTNAFR	576.811647	2
sp|P05164|PERM_HUMAN	IANVFTNAFR	581.815782	2
sp|P05164|PERM_HUMAN	VVLEGGIDPILR	640.88227	2
sp|P05164|PERM_HUMAN	VVLEGGIDPILR	645.886404	2
sp|P14174|MIF_HUMAN	LLCGLLAER	522.797151	2
sp|P14174|MIF_HUMAN	LLCGLLAER	527.801286	2
sp|P14174|MIF_HUMAN	VYINYYDMNAANVGWNNSTFA	809.695191	3
sp|P14174|MIF_HUMAN	VYINYYDMNAANVGWNNSTFA	813.037601	3
sp|P01833|PIGR_HUMAN	GGCITLISSEGYVSSK	829.40872	2
sp|P01833|PIGR_HUMAN	GGCITLISSEGYVSSK	833.415819	2
sp|P01833|PIGR_HUMAN	VYTVDLGR	461.753267	2
sp|P01833|PIGR_HUMAN	VYTVDLGR	466.757401	2
sp|P05787|K2C8_HUMAN	WSLLQQQK	515.787641	2
sp|P05787|K2C8_HUMAN	WSLLQQQK	519.79474	2
sp|P05787|K2C8_HUMAN	LSELEAALQR	565.31422	2
sp|P05787|K2C8_HUMAN	LSELEAALQR	570.318354	2
sp|O43278|SPIT1_HUMAN	WYYDPTEQICK	751.834659	2
sp|O43278|SPIT1_HUMAN	WYYDPTEQICK	755.841759	2
sp|O43278|SPIT1_HUMAN	SFVYGGCLGNK	601.287148	2
sp|O43278|SPIT1_HUMAN	SFVYGGCLGNK	605.294247	2
sp|P06733|ENOA_HUMAN	TIAPALVSK	450.28166	2
sp|P06733|ENOA_HUMAN	TIAPALVSK	454.28876	2
sp|P06733|ENOA_HUMAN	YISPDQLADLYK	713.366649	2
sp|P06733|ENOA_HUMAN	YISPDQLADLYK	717.373749	2
sp|Q16658|FSCN1_HUMAN	FLIVAHDDGR	381.536612	3
sp|Q16658|FSCN1_HUMAN	FLIVAHDDGR	384.872701	3
sp|Q16658|FSCN1_HUMAN	YLAPSGPSGTLK	595.82442	2
sp|Q16658|FSCN1_HUMAN	YLAPSGPSGTLK	599.83152	2
sp|P43490|NAMPT_HUMAN	YDGHLPIEIK	395.548517	3
sp|P43490|NAMPT_HUMAN	YDGHLPIEIK	398.219917	3
sp|P43490|NAMPT_HUMAN	YLLETSGNLDGLEYK	857.930342	2
sp|P43490|NAMPT_HUMAN	YLLETSGNLDGLEYK	861.937442	2
sp|P40121|CAPG_HUMAN	YQEGGVESAFHK	451.214086	3
sp|P40121|CAPG_HUMAN	YQEGGVESAFHK	453.885486	3
sp|P40121|CAPG_HUMAN	EGNPEEDLTADK	659.293878	2
sp|P40121|CAPG_HUMAN	EGNPEEDLTADK	663.300978	2
