# Supplementary material for: Cervical Fluids Are a Source of Protein Biomarkers for Early, Non-Invasive Endometrial Cancer Diagnosis
Source: Cancers (Basel). 2023 Jan 31;15(3):911. doi: 10.3390/cancers15030911 (PMC9913506; doi:10.3390/cancers15030911)
Supplement: Supplementary file 1 [file cancers-15-00911-s001.zip › Suppl_Table2.2.pdf]

P04083	ANXA1	DITSDTSGDFR	**6.761**	**0.011**	**0.7745**	0.698	0.484	0.592	1.122	0.958	0.507
P09211	GSTP1	TLGLYGK	**2.910**	**0.011**	**0.7745**	1.277	0.269	0.650	1.372	**0.048**	**0.725**
P30041	PRDX6	LSILYPATTGR	**3.283**	**0.014**	**0.7647**	1.326	0.269	0.647	1.434	0.077	0.696
P14174	MIF	VYINYYDMNAANVGWNNSTFA	**8.416**	**0.008**	**0.7598**	1.339	0.210	0.681	1.524	0.053	**0.719**
O43852	CALU	TFDQLTPEESK	**4.042**	**0.016**	**0.7582**	**2.083**	0.128	**0.725**	1.578	0.082	0.691
P07237	P4HB	ALAPEYAK	**2.741**	**0.016**	**0.7582**	0.780	0.777	0.536	1.117	0.573	0.562
Q13938	CAPS	SGDGVVTVDDLR	**3.670**	**0.018**	**0.7549**	**2.351**	0.210	0.681	1.409	0.437	0.583
P22626	HNRNPA2B1	TLETVPLER	**2.214**	**0.020**	**0.7500**	1.412	0.269	0.657	1.536	0.059	**0.712**
P10451	SPP1	AIPVAQDLNAPSDWDSR	**3.958**	**0.021**	**0.7484**	0.851	0.226	0.675	0.580	0.766	0.533
Q13938	CAPS	EAVIAAAFAK	**3.323**	**0.024**	**0.7418**	**2.136**	0.248	0.667	1.631	0.299	0.614
P50454	SERPINH1	GVVEVTHDLQK	**2.413**	**0.026**	**0.7386**	**5.886**	0.062	**0.828**	**3.977**	**0.038**	**0.735**
Q01995	TAGLN	TLMALGSLAVTK	**2.046**	**0.040**	**0.7222**	**3.938**	0.269	0.652	**6.348**	**0.016**	**0.840**
P30086	PEBP1	VLTPTQVK	1.967	**0.040**	**0.7222**	0.766	0.844	0.523	1.392	0.071	**0.703**
P80188	LCN2	VPLQQNFQDNQFQGK	**2.535**	**0.044**	**0.7157**	0.661	0.270	0.644	0.648	0.190	0.647
P30086	PEBP1	LYEQLSGK	1.997	**0.045**	**0.7157**	0.944	0.720	0.549	1.410	0.077	0.696
P50454	SERPINH1	DTQSGSLLFIGR	**2.674**	**0.042**	**0.7157**	**5.425**	0.210	0.662	**4.222**	**0.046**	**0.717**
P05787	KRT8	LSELEAALQR	**12.502**	**0.047**	**0.7124**	**5.547**	0.114	**0.761**	**2.612**	**0.036**	**0.742**
P17931	LGALS3	GNDVAFHFNPR	**2.540**	**0.047**	**0.7124**	1.239	0.300	0.627	1.322	**0.018**	**0.797**
P22626	HNRNPA2B1	GGNFGFGDSR	**2.706**	**0.047**	**0.7108**	1.343	0.278	0.637	1.989	**0.021**	**0.775**
P17931	LGALS3	IALDFQR	**2.606**	0.057	**0.7026**	1.274	0.278	0.641	1.283	**0.018**	**0.794**
P09466	PAEP	VLVEDDEIMQGFIR	0.038	**0.047**	0.6993	0.007	0.248	0.660	0.003	0.054	**0.712**
P07339	CTSD	VGFAEAAR	1.575	0.069	0.6928	1.485	0.270	0.644	1.166	0.100	0.680
P07339	CTSD	VSTLPAITLK	1.598	0.069	0.6928	1.217	0.637	0.569	1.104	0.233	0.634
P80188	LCN2	ELTSELK	**2.045**	0.074	0.6895	0.631	0.278	0.637	0.671	0.233	0.634
Q16658	FSCN1	YLAPSGPSGTLK	1.800	0.084	0.6830	1.463	0.300	0.631	**2.187**	**0.034**	**0.745**
P07355	ANXA2	QDIAFAYQR	**8.887**	0.066	0.6797	0.719	0.827	0.526	1.114	0.097	0.681
Q01995	TAGLN	EFTESQLQEGK	1.881	0.111	0.6699	**5.254**	0.269	0.641	**5.804**	**0.021**	**0.773**
Q16658	FSCN1	FLIVAHDDGR	1.960	0.122	0.6634	1.865	0.154	**0.709**	**2.124**	**0.048**	**0.724**
P05787	KRT8	WSLLQQQK	**13.174**	0.122	0.6634	**6.437**	0.114	**0.742**	**2.400**	**0.048**	**0.725**
P08253	MMP2	VDAAFNWSK	0.727	0.121	0.6634	1.664	0.398	0.608	0.716	0.088	0.688
P15941	MUC1	EGTINVHDVETQFNQYK	1.751	0.131	0.6585	0.899	0.918	0.511	1.682	0.082	0.693
P15941	MUC1	QGGFLGLSNIK	1.785	0.181	0.6405	1.015	0.656	0.565	1.413	0.190	0.647
P09382	LGALS1	LPDGYEFK	1.368	0.181	0.6405	**2.074**	0.114	**0.745**	**2.030**	**0.044**	**0.732**
P14618	PKM	APIIAVTR	1.926	0.181	0.6405	**4.230**	0.269	0.654	1.492	0.212	0.641
P00441	SOD1	AVCVLK	1.631	0.181	0.6405	0.936	0.827	0.526	1.384	0.091	0.686
P00441	SOD1	TLVVHEK	1.620	0.190	0.6373	0.888	0.702	0.552	1.382	0.114	0.673
P09382	LGALS1	SFVLNLGK	1.249	0.365	0.5980	**2.018**	0.131	**0.729**	**2.099**	**0.026**	**0.761**
P01833	PIGR	GGCITLISSEGYVSSK	1.127	0.468	0.5801	0.265	0.210	0.683	0.661	0.290	0.618
Q14508	WFDC2	VSCVTPNF	0.900	0.611	0.5588	0.514	0.656	0.562	0.519	0.096	0.683
P15328	FOLR1	FNWNHCGEMAPACK	0.362	0.628	0.5556	0.557	0.765	0.539	0.530	0.282	0.621
P09603	CSF1	TFYETPLQLLEK	0.917	0.678	0.5458	1.302	0.777	0.533	1.330	0.290	0.614
P01833	PIGR	VYTVDLGR	1.152	0.739	0.5392	0.547	0.475	0.595	0.673	0.311	0.611
P08670	VIM	ILLAELEQLK	0.994	0.757	0.5359	1.189	0.269	0.647	1.485	0.075	0.699
P15328	FOLR1	TELLNVCMNAK	0.399	0.759	0.5343	0.591	0.974	0.503	0.605	0.347	0.601
P16035	TIMP2	EVDSGNDIYGNPIK	0.876	0.805	0.5278	0.563	0.278	0.637	0.660	0.077	0.696
P08670	VIM	ISLPLPNFSSLNLR	0.965	0.861	0.5196	**2.646**	0.095	**0.804**	**2.722**	**0.019**	**0.784**
O75556	SCGB2A1	TINSDISIPEYK	**2.476**	0.930	0.5098	0.188	0.827	0.526	0.134	0.835	0.523
Q14508	WFDC2	EGSCPQVNINFPQLGLCR	1.118	0.961	0.5049	0.517	0.765	0.539	0.529	0.299	0.614
