# Supplementary material for: Cervical Fluids Are a Source of Protein Biomarkers for Early, Non-Invasive Endometrial Cancer Diagnosis
Source: Cancers (Basel). 2023 Jan 31;15(3):911. doi: 10.3390/cancers15030911 (PMC9913506; doi:10.3390/cancers15030911)
Supplement: Supplementary file 1 [file cancers-15-00911-s001.zip › Suppl_Table2.pdf]

			UTERINE FLUIDS			M1 CERVICAL SAMPLES			M3 CERVICAL SAMPLES		
Entry	Gene Name	Peptide	Fold Change	FDR	AUC	Fold Change	FDR	AUC	Fold Change	FDR	AUC
P00338	LDHA	LVIITAGAR	7.127	0.000	0.9575	1.486	0.114	**0.758**	1.572	**0.034**	**0.748**
P06733	ENO1	YISPDQLADLYK	5.091	0.000	0.9477	1.209	0.210	0.690	1.690	**0.016**	**0.814**
P00338	LDHA	VTLTSEEEAR	6.183	0.000	0.9444	1.471	0.114	**0.758**	1.502	**0.041**	**0.735**
P06733	ENO1	TIAPALVSK	4.753	0.000	0.9379	1.322	0.154	**0.716**	1.561	**0.018**	**0.791**
P14618	PKM	NTGIICTIGPASR	5.405	0.000	0.9346	1.317	0.211	0.680	1.374	**0.019**	**0.781**
P42574	CASP3	SGTDVDAANLR	6.428	0.001	0.9020	1.410	0.251	0.663	1.250	0.059	**0.712**
P16949	STMN1	SHEAEVLK	4.651	0.001	0.8987	1.491	0.154	**0.711**	**2.318**	**0.022**	**0.770**
O00299	CLIC1	LAALNPESNTAGLDIFAK	3.373	0.001	0.8922	1.125	0.300	0.627	1.694	**0.018**	**0.801**
O00299	CLIC1	NSNPALNDNLEK	3.290	0.001	0.8922	1.244	0.251	0.663	1.422	**0.021**	**0.771**
P55060	CSE1L	ANIVHLMLSSPEQIQK	5.637	0.001	0.8922	**2.013**	0.114	**0.765**	**2.248**	**0.016**	**0.827**
P55060	CSE1L	LLQTDDEEEAGLLELLK	5.914	0.001	0.8905	**2.165**	0.095	**0.788**	**2.527**	**0.016**	**0.819**
P14174	MIF	LLCGLLAER	3.669	0.001	0.8889	1.229	0.269	0.647	1.628	**0.018**	**0.797**
P43490	NAMPT	YLLETSGNLDGLEYK	10.476	0.001	0.8889	1.664	0.154	**0.711**	1.183	0.259	0.627
Q06830	PRDX1	LVQAFQFTDK	5.115	0.001	0.8889	1.344	0.248	0.667	1.565	**0.016**	**0.807**
P16949	STMN1	AIEENNNFSK	4.630	0.001	0.8889	1.582	0.154	**0.712**	**2.319**	**0.021**	**0.771**
Q01469	FABP5	LVVECVMNNVTCTR	2.730	0.001	0.8856	0.376	0.738	0.546	1.104	0.723	0.539
Q01469	FABP5	ELGVGIALR	2.544	0.001	0.8824	0.460	0.765	0.539	1.004	0.974	0.503
O43278	SPINT1	SFVYGGCLGNK	4.416	0.001	0.8824	0.960	0.666	0.559	1.528	**0.046**	**0.729**
P43490	NAMPT	YDGHLPIEIK	9.572	0.001	0.8725	1.739	0.114	**0.748**	1.074	0.324	0.608
P12830	CDH1	VFYSITGQGADTPPVGVFIIER	4.498	0.001	0.8660	0.775	0.619	0.572	1.832	**0.018**	**0.791**
O43278	SPINT1	WYYDPTEQICK	4.056	0.001	0.8644	0.755	0.666	0.559	1.527	0.071	**0.703**
P61604	HSPE1	FLPLFDR	5.020	0.002	0.8529	1.986	0.134	**0.725**	1.987	**0.019**	**0.781**
P35222	CTNNB1	LVQLLVR	4.875	0.002	0.8480	**2.034**	0.114	**0.748**	**2.129**	**0.016**	**0.819**
Q06830	PRDX1	ADEGISFR	4.575	0.002	0.8448	1.449	0.210	0.693	1.586	**0.016**	**0.817**
P12830	CDH1	NLVQIK	3.738	0.002	0.8382	0.897	0.888	0.516	1.817	**0.016**	**0.814**
P62937	PPIA	FEDENFILK	2.830	0.002	0.8366	1.142	0.308	0.624	1.582	**0.019**	**0.784**
P60174	TPI1	VVLAYEPVWAIGTGK	3.320	0.003	0.8333	1.268	0.278	0.637	1.664	**0.019**	**0.781**
P62937	PPIA	VSFELFADK	3.057	0.003	0.8301	1.178	0.290	0.634	1.835	**0.016**	**0.833**
P61604	HSPE1	VLLPEYGGTK	5.448	0.003	0.8268	1.744	0.210	0.683	1.871	**0.027**	**0.758**
P40121	CAPG	EGNPEEDLTADK	4.366	0.003	0.8268	1.358	0.308	0.624	1.389	0.065	**0.708**
P14780	MMP9	SLGPALLLLQK	3.551	0.003	0.8235	0.670	0.475	0.595	0.439	0.270	0.624
P07237	P4HB	ILEFFGLK	3.264	0.004	0.8186	0.913	0.300	0.627	1.249	0.338	0.605
P35222	CTNNB1	LLNDEDQVVVNK	4.478	0.004	0.8137	**2.069**	0.210	0.683	**2.318**	**0.019**	**0.784**
P40121	CAPG	YQEGGVESAFHK	3.808	0.004	0.8121	1.403	0.270	0.644	1.404	**0.027**	**0.758**
P10451	SPP1	ANDESNEHSDVIDSQELSK	8.453	0.004	0.8105	1.961	0.114	**0.745**	1.404	0.595	0.557
P16070	CD44	TEAADLCK	3.198	0.005	0.8072	1.712	0.131	**0.729**	1.623	**0.034**	**0.745**
P05164	MPO	VVLEGGIDPILR	9.227	0.006	0.8007	1.363	0.702	0.552	1.078	0.347	0.601
P07355	ANXA2	GVDEVTIVNILTNR	8.099	0.006	0.7974	0.682	0.656	0.562	1.347	**0.034**	**0.745**
P14780	MMP9	AFALWSAVTPLTFTR	3.671	0.006	0.7974	0.647	0.501	0.588	0.583	0.417	0.588
P05164	MPO	IANVFTNAFR	8.567	0.007	0.7941	1.498	0.656	0.562	0.953	0.590	0.559
P04792	HSPB1	LATQSNEITIPVTFESR	5.090	0.007	0.7908	1.534	0.269	0.650	1.325	**0.016**	**0.824**
P60174	TPI1	SNVSDAVAQSTR	3.397	0.007	0.7908	1.369	0.210	0.690	1.510	**0.024**	**0.765**
P30041	PRDX6	DFTPVCTTELGR	2.831	0.009	0.7843	1.234	0.300	0.627	1.645	**0.034**	**0.748**
P09211	GSTP1	ASCLYGQLPK	2.951	0.009	0.7843	1.274	0.269	0.650	1.293	0.059	**0.712**
P04792	HSPB1	LFDQAFGLPR	5.632	0.010	0.7778	1.742	0.262	0.660	1.235	**0.016**	**0.810**
P04083	ANXA1	GGPGSAVSPYPTFNPSSDVAALHK	6.153	0.011	0.7745	0.622	0.484	0.592	1.265	0.432	0.585

**P04083**	**ANXA1**	**DITSDTSGDFR**	**6.761**	**0.011**	**0.7745**	0.698	0.484	0.592	1.122	0.958	0.507
**P09211**	**GSTP1**	**TLGLYGK**	**2.910**	**0.011**	**0.7745**	1.277	0.269	0.650	1.372	**0.048**	**0.725**
**P30041**	**PRDX6**	**LSILYPATTGR**	**3.283**	**0.014**	**0.7647**	1.326	0.269	0.647	1.434	0.077	0.696
**P14174**	**MIF**	**VYINYYDMNAANVGWNNSTFA**	**8.416**	**0.008**	**0.7598**	1.339	0.210	0.681	1.524	0.053	**0.719**
**O43852**	**CALU**	**TFDQLTPEESK**	**4.042**	**0.016**	**0.7582**	**2.083**	0.128	**0.725**	1.578	0.082	0.691
**P07237**	**P4HB**	**ALAPEYAK**	**2.741**	**0.016**	**0.7582**	0.780	0.777	0.536	1.117	0.573	0.562
**Q13938**	**CAPS**	**SGDGVVTVDDLR**	**3.670**	**0.018**	**0.7549**	**2.351**	0.210	0.681	1.409	0.437	0.583
**P22626**	**HNRNPA2B1**	**TLETVPLER**	**2.214**	**0.020**	**0.7500**	1.412	0.269	0.657	1.536	0.059	**0.712**
**P10451**	**SPP1**	**AIPVAQDLNAPSDWDSR**	**3.958**	**0.021**	**0.7484**	0.851	0.226	0.675	0.580	0.766	0.533
**Q13938**	**CAPS**	**EAVIAAAFAK**	**3.323**	**0.024**	**0.7418**	**2.136**	0.248	0.667	1.631	0.299	0.614
**P50454**	**SERPINH1**	**GVVEVTHDLQK**	**2.413**	**0.026**	**0.7386**	**5.886**	0.062	**0.828**	**3.977**	**0.038**	**0.735**
**Q01995**	**TAGLN**	**TLMALGSLAVTK**	**2.046**	**0.040**	**0.7222**	**3.938**	0.269	0.652	**6.348**	**0.016**	**0.840**
**P30086**	**PEBP1**	**VLTPTQVK**	1.967	**0.040**	**0.7222**	0.766	0.844	0.523	1.392	0.071	**0.703**
**P80188**	**LCN2**	**VPLQQNFQDNQFQGK**	**2.535**	**0.044**	**0.7157**	0.661	0.270	0.644	0.648	0.190	0.647
**P30086**	**PEBP1**	**LYEQLSGK**	1.997	**0.045**	**0.7157**	0.944	0.720	0.549	1.410	0.077	0.696
**P50454**	**SERPINH1**	**DTQSGSLLFIGR**	**2.674**	**0.042**	**0.7157**	**5.425**	0.210	0.662	**4.222**	**0.046**	**0.717**
**P05787**	**KRT8**	**LSELEAALQR**	**12.502**	**0.047**	**0.7124**	**5.547**	0.114	**0.761**	**2.612**	**0.036**	**0.742**
**P17931**	**LGALS3**	**GNDVAFHFNPR**	**2.540**	**0.047**	**0.7124**	1.239	0.300	0.627	1.322	**0.018**	**0.797**
**P22626**	**HNRNPA2B1**	**GGNFGFGDSR**	**2.706**	**0.047**	**0.7108**	1.343	0.278	0.637	1.989	**0.021**	**0.775**
**P17931**	**LGALS3**	**IALDFQR**	**2.606**	0.057	**0.7026**	1.274	0.278	0.641	1.283	**0.018**	**0.794**
**P09466**	**PAEP**	**VLVEDDEIMQGFIR**	0.038	**0.047**	0.6993	0.007	0.248	0.660	0.003	0.054	**0.712**
**P07339**	**CTSD**	**VGFAEAAR**	1.575	0.069	0.6928	1.485	0.270	0.644	1.166	0.100	0.680
**P07339**	**CTSD**	**VSTLPAITLK**	1.598	0.069	0.6928	1.217	0.637	0.569	1.104	0.233	0.634
**P80188**	**LCN2**	**ELTSELK**	**2.045**	0.074	0.6895	0.631	0.278	0.637	0.671	0.233	0.634
**Q16658**	**FSCN1**	**YLAPSGPSGTLK**	1.800	0.084	0.6830	1.463	0.300	0.631	**2.187**	**0.034**	**0.745**
**P07355**	**ANXA2**	**QDIAFAYQR**	**8.887**	0.066	0.6797	0.719	0.827	0.526	1.114	0.097	0.681
**Q01995**	**TAGLN**	**EFTESQLQEGK**	1.881	0.111	0.6699	**5.254**	0.269	0.641	**5.804**	**0.021**	**0.773**
**Q16658**	**FSCN1**	**FLIVAHDDGR**	1.960	0.122	0.6634	1.865	0.154	**0.709**	**2.124**	**0.048**	**0.724**
**P05787**	**KRT8**	**WSLLQQQK**	**13.174**	0.122	0.6634	**6.437**	0.114	**0.742**	**2.400**	**0.048**	**0.725**
**P08253**	**MMP2**	**VDAAFNWSK**	0.727	0.121	0.6634	1.664	0.398	0.608	0.716	0.088	0.688
**P15941**	**MUC1**	**EGTINVHDVETQFNQYK**	1.751	0.131	0.6585	0.899	0.918	0.511	1.682	0.082	0.693
**P15941**	**MUC1**	**QGGFLGLSNIK**	1.785	0.181	0.6405	1.015	0.656	0.565	1.413	0.190	0.647
**P09382**	**LGALS1**	**LPDGYEFK**	1.368	0.181	0.6405	**2.074**	0.114	**0.745**	**2.030**	**0.044**	**0.732**
**P14618**	**PKM**	**APIIAVTR**	1.926	0.181	0.6405	**4.230**	0.269	0.654	1.492	0.212	0.641
**P00441**	**SOD1**	**AVCVLK**	1.631	0.181	0.6405	0.936	0.827	0.526	1.384	0.091	0.686
**P00441**	**SOD1**	**TLVVHEK**	1.620	0.190	0.6373	0.888	0.702	0.552	1.382	0.114	0.673
**P09382**	**LGALS1**	**SFVLNLGK**	1.249	0.365	0.5980	**2.018**	0.131	**0.729**	**2.099**	**0.026**	**0.761**
**P01833**	**PIGR**	**GGCITLISSEGYVSSK**	1.127	0.468	0.5801	0.265	0.210	0.683	0.661	0.290	0.618
**Q14508**	**WFDC2**	**VSCVTPNF**	0.900	0.611	0.5588	0.514	0.656	0.562	0.519	0.096	0.683
**P15328**	**FOLR1**	**FNWNHCGEMAPACK**	0.362	0.628	0.5556	0.557	0.765	0.539	0.530	0.282	0.621
**P09603**	**CSF1**	**TFYETPLQLLEK**	0.917	0.678	0.5458	1.302	0.777	0.533	1.330	0.290	0.614
**P01833**	**PIGR**	**VYTVDLGR**	1.152	0.739	0.5392	0.547	0.475	0.595	0.673	0.311	0.611
**P08670**	**VIM**	**ILLAELEQLK**	0.994	0.757	0.5359	1.189	0.269	0.647	1.485	0.075	0.699
**P15328**	**FOLR1**	**TELLNVCMNAK**	0.399	0.759	0.5343	0.591	0.974	0.503	0.605	0.347	0.601
**P16035**	**TIMP2**	**EVDSGNDIYGNPIK**	0.876	0.805	0.5278	0.563	0.278	0.637	0.660	0.077	0.696
**P08670**	**VIM**	**ISLPLPNFSSLNLR**	0.965	0.861	0.5196	**2.646**	0.095	**0.804**	**2.722**	**0.019**	**0.784**
**O75556**	**SCGB2A1**	**TINSDISIPEYK**	**2.476**	0.930	0.5098	0.188	0.827	0.526	0.134	0.835	0.523
**Q14508**	**WFDC2**	**EGSCPQVNINFPQLGLCR**	1.118	0.961	0.5049	0.517	0.765	0.539	0.529	0.299	0.614
