# Supplementary figures and images for: Cervical Fluids Are a Source of Protein Biomarkers for Early, Non-Invasive Endometrial Cancer Diagnosis
Source: Cancers (Basel). 2023 Jan 31;15(3):911. doi: 10.3390/cancers15030911 (PMC9913506; doi:10.3390/cancers15030911)

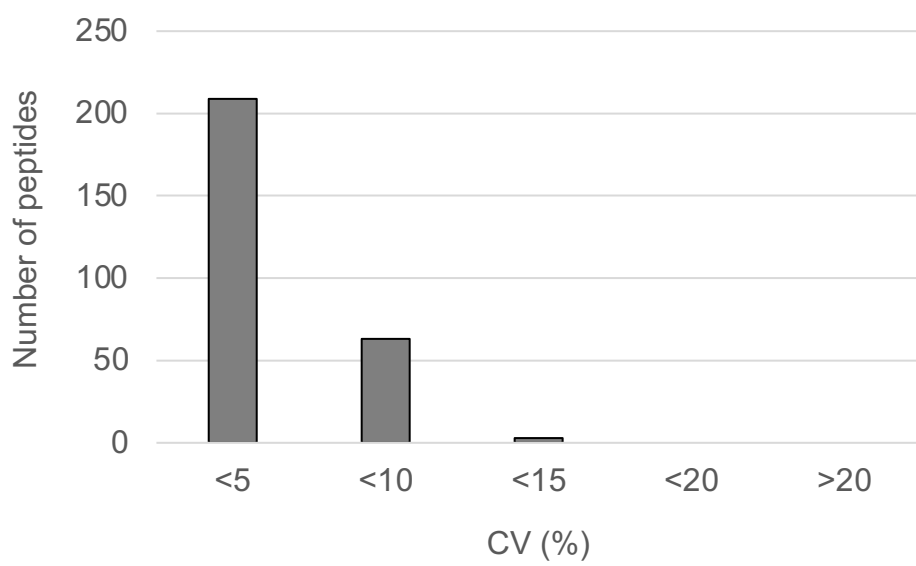

Supplement: Supplementary file 1 [file cancers-15-00911-s001.zip › Suppl_Figure1.pdf]

## A M1 Cervical fluids

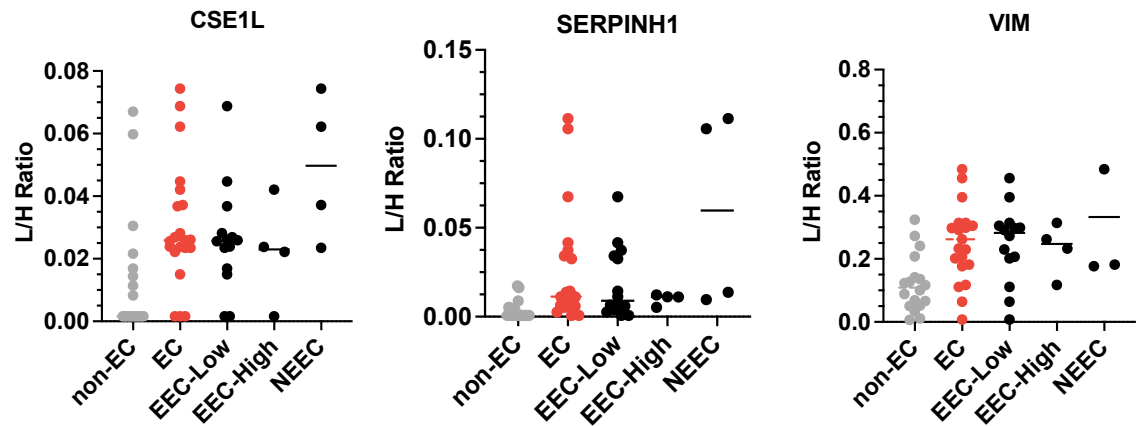

## B M3 Cervical fluids

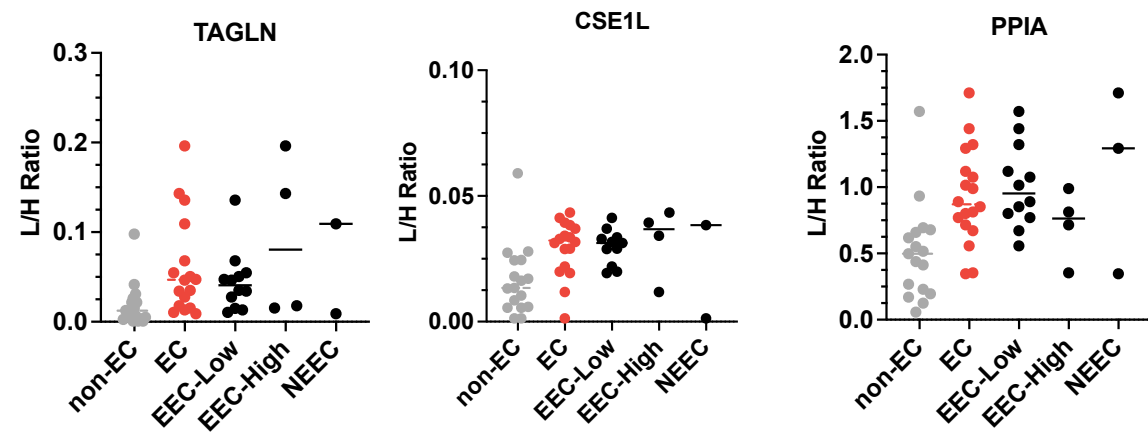

Supplement: Supplementary file 1 [file cancers-15-00911-s001.zip › Suppl_Figure2.pdf]
